# Quantitative and Semi-quantitative Methods for Assessing the Degree of Methylene Blue Staining in Sentinel Lymph Nodes in Dogs

**DOI:** 10.3389/fvets.2021.758295

**Published:** 2021-10-21

**Authors:** Ann S. Ram, Kathy Matuszewska, Jim Petrik, Ameet Singh, Michelle L. Oblak

**Affiliations:** ^1^Department of Clinical Studies, Ontario Veterinary College, University of Guelph, Guelph, ON, Canada; ^2^Department of Biomedical Sciences, Ontario Veterinary College, University of Guelph, Guelph, ON, Canada

**Keywords:** methylene blue, sentinel lymph node mapping, image analysis, semi-quantitative scoring, staining

## Abstract

**Background:** To develop a digital algorithm for quantitative assessment of surface methylene blue staining in whole lymph nodes and validate a semi-quantitative visual scoring method for patient-side use.

**Methods:** Lymph nodes from canine patients with spontaneous tumors undergoing sentinel lymph node mapping were prospectively assessed *ex vivo* and photographed. Using an open-source computer-based imaging software, an algorithm was developed for quantification of staining based on a signal-to-background ratio. Next, two blinded observers evaluated images and assigned a semi-quantitative visual score based on surface staining (0—no blue stain, 1−1–50% stained, and 2−51–100% stained) and those results were compared to the established quantitative standard.

**Results:** Forty-three lymph nodes were included. Image analysis successfully quantified blue staining and differentiated from normal lymph node tissue in all cases. Agreement between observers using the Kappa coefficient demonstrated strong agreement (*k* = 0.8581, *p* < 0.0001) between semi-quantitative visual scoring and image analysis. There was substantial interobserver and intraobserver agreement for the scoring system (*k* = 0.7340, *p* < 0.0001 and *k* = 0.8983, *p* < 0.0001, respectively).

**Conclusion:** A digital algorithm using an open-source software was simple and straightforward to use for quantification of blue staining. The use of a semi-quantitative visual scoring system shows promise for a simple, objective, repeatable assessment of methylene blue staining at the time of surgery. This study demonstrates reliable and repeatable methods for blue staining quantification thereby providing a novel and objective reporting mechanism in scientific research involving sentinel lymph node mapping.

## Introduction

Sentinel lymph nodes (SLN) represent the primary site of solid tumor drainage and are valuable indicators for cancer staging and treatment recommendations (1, 2). Detecting SLNs is achieved by using lymphatic tracers, most commonly injected peritumorally, that delineate the sentinel node(s) (3). In human medicine, the gold standard in SLN mapping employs dual tracer techniques involving a combination of radioisotopes, blue dyes and/or fluorescence to increase reliability (4–7). However, in specific countries and facilities, methylene blue dye is used alone for SLN mapping in light of its cost effectiveness, accessibility, and safe outcomes (4, 8, 9). Methylene blue is a non-specific blue dye with a good safety profile and has been described for use as an alternative to isosulfan blue and patent blue dye (2, 4, 10). Breast cancer studies that employ methylene blue dye alone suggest comparable lymphatic uptake and results to other blue dyes (2, 11–15).

Typically, human and veterinary studies utilizing methylene blue report a subjective assessment of the presence or absence of blue staining (16–19). While this information may be adequate clinically, when reporting in research studies, a more objective method for determining both the presence and degree of blue staining is necessary. ImageJ is an open-source, widely available software that allows for quantification of histological staining and immunofluorescence of microscopy images (20, 21). Protocols for methylene blue stain analysis is not yet distinguished for whole organ specimens, however, ImageJ is utilized to detect methylene blue in cellular microscopy (22) or tissue staining (23). ImageJ detects staining, such as those for methylene blue and a similar dye called toluidine blue in applications relating to cellular staining and assays (24–27). Based on the ImageJ user guide, detecting blue hues is straightforward and stringent, specifically when colors are convoluted or observers are color-blind (28).

The focal challenge with SLN mapping studies utilizing methylene blue is the lack of standardization in reporting and objective inclusion criteria for discerning blue and non-blue stained surgical specimens. Whether, methylene blue is used alone or in combination with other imaging modalities, reporting must be stringent and reproducible between studies to accurately evaluate and compare the blue dye and the techniques employed. To improve methylene blue SLN mapping research, either the absence of distinct inclusion criteria must be avoided or the removal of subjective evaluation methods that are not translatable between studies or investigators.

Minor challenges associated with the use of methylene blue in SLN mapping, can be the correct identification of blue staining compared to normal surface coloration. Lymph nodes are often heterogenous in morphology (29) and brown tissue can appear as blue under certain circumstances. The discernment of blue stain on a lymph node becomes difficult in cases where limited uptake occurs as the clinician cannot identify whether the discoloration is due to staining or to natural lymph node tissue pigmentation. In cases where a dyed lymphatic is not visible, this challenge can pose some difficulty to the clinician in confirming that a lymph node is truly sentinel when methylene blue is used alone.

In a surgical setting, a digital algorithm is not practical nor efficient to assess the lymph node in real time. Therefore, an objective, accessible method for methylene blue stain quantification on whole tissue at the patient side is needed to improve reporting. Semi-quantitative scoring systems have been reported for immunohistochemistry of stained tissues (30, 31). We will utilize similar methodology for intraoperative scoring of blue staining on the surface of lymph nodes.

The purpose of this study is to develop a digital algorithm for quantitative assessment of surface methylene blue staining in whole lymph nodes using an open-access program for image analysis and to validate a semi-quantitative visual scoring method for patient-side use. We hypothesize that the use of an open-source imaging software will provide a straightforward and accessible method for objective scoring of blue surface staining and there will be good agreement between scoring and analysis results to justify the validity of the semi-quantitative visual assessment.

## Materials and Methods

### Imaging of Lymph Nodes Stained With Methylene Blue

Lymph nodes were obtained consecutively from canine patients with spontaneous tumors of various sites undergoing lymph node extirpation as part of a concurrent SLN biopsy study at the Ontario Veterinary College Health Sciences Center from 2017 to 2019 (Supplementary Table 1). In all patients, a 1 mL solution of methylene blue was injected peritumorally at a concentration of 0.5 mg/mL (19, 32) and routine regional lymph node extirpation performed. Lymph nodes were imaged in either an unstandardized or standardized fashion, depending on which part of the accrual period they were removed. For the unstandardized group, imaging of lymph nodes was performed with an iPhone 6 or newer model, equipped with a 12 mega-pixel camera (Apple, California, USA) in an unstandardized fashion with no lighting, background, or camera distance controls. For the standardized group, lymph nodes were placed on a uniform white background in a photo lightbox (Amazon, Canada) equipped with LED lights to provide optimal imaging conditions for gross specimens (33, 34). Using an iPhone X equipped with a 12-megapixel wide angle camera and secondary telephoto lens (Apple, California, USA) images were taken at a distance of 20 cm from the specimen through a 1 cm hole at the top of the box to improve focus of the smartphone lens.

### Semi-quantitative Visual Scoring of Methylene Blue Stain

All lymph nodes were assessed for methylene blue staining by the primary investigator (M.O) *in situ* providing an intraoperative score before extirpation and lymph nodes considered positive for blue staining (sentinel) were included in this study. Inclusion criteria of lymph nodes was based on the surgeon's identification of the node being positive for blue staining. Negative control lymph nodes obtained from routine biopsies were also included. Immediately after removal, lymph nodes were photographed and assigned a postoperative *ex vivo* score based on the coloration of the surface of the lymph node by a blinded investigator (A.R). The intra and postoperative scoring data was collected *via* surgical sheet recordings with the two investigators blinded to each other's score. The data was then input into the data collection sheet for statistical analysis. Evaluation by each observer consisted of assessment of the degree of blue surface staining based on a semi-quantitative visual scoring system. Scores were assigned as follows: 0 = no blue stain, 1+ = 1–50% staining of the surface of the lymph node, 2+ = 51–100% staining of the surface of the lymph node. After completion of the data collection phase of the study, all images were randomized for evaluation to assess for both inter and intraobserver agreement. At least 4 weeks was allowed to pass, and the investigators were blinded to the original findings. The randomized images were then evaluated and scored by 2 investigators (M.O., A.R.) for blue stain visualized on the surface of the lymph node.

### Quantitative Image Analysis Validation

Images were processed and analyzed in FIJI (National Institutes of Health, Bethesda, Maryland, USA), a distribution of an open-source image processing program (ImageJ) (35) with bioscience centered plugins. Software parameters were defined with negative and positive control lymph nodes images (36). Negative controls were cases of clinically normal lymph nodes from routine biopsies with no methylene blue exposure. Thresholds were adjusted using negative controls and utilizing a threshold of 0–125 after running the deconvolution plug-in on negative control images depicted 0% of stain detected for all controls (37). Positive controls were cases where a highly stained region of the lymph node could be cropped to areas of stain only; in the form of a region-of-interest (ROI) (38). Validation to detect methylene blue staining on whole tissue specimens and determining the threshold to differentiate dark blue staining from false-positive signals was completed with the positive controls and >95% signal detection was obtained with the 0–125 threshold.

### Quantification of Methylene Blue Stain Using Image Analysis

Images were analyzed in two randomized groups of 21 and 22; consisting of 43 images analyzed in total. Color and background corrections were performed on the true color (RGB) image using the “Subtract Background” feature and auto adjustment function of the “Brightness/Contrast” tool (36). By default, the area was measured in pixels. A ROI was drawn around the whole lymph node using the “Color Threshold” function, producing an automatic threshold over the image that was measured as the area of the entire surface of the lymph node (39). If auto threshold did not accurately differentiate the area of the lymph node from the image background, the thresholding brightness and saturation levels were adjusted (36). Subsequent use of the Color Deconvolution plugin on the RGB image separated pigments into channels (35). Following image processing there was an output of 3 channels in 8-bit grayscale format. The “Color_1” window corresponding to the methylene blue channel was used for analysis (40). Thresholding using the 8-bit image and a threshold of 0–125 resulted in a binary image of the area of MB staining. The amount of MB stain on the surface of the node was then calculated as a percentage using the following equation:


$$\begin{array}{l} \frac{Area\;of\;MB\;stain}{Area\;of\;lymph\;node}\times 100 \end{array}$$

### Statistical Analysis

All analyses were completed using SAS 9.3 by a biostatistician who was blinded to the clinical procedures and assessment protocols. Descriptive analysis was performed with summaries of frequencies and agreement percentages in contingency tables. Quantitative image analysis was designated as the gold standard. Weighted kappa (κ) statistics were used to assess agreement of assessment modalities, inter- and intra-observer agreement, and agreement between scoring settings. Weighted kappa values with 95% confidence intervals (CI) were calculated to determine strength of agreement. Coefficients in the range of 0.21–0.40 were interpreted as fair agreement, 0.41–0.60 as moderate agreement, 0.61–0.80 as substantial agreement, and 0.81–1 as almost perfect agreement (41). Statistical significance was set at a two-sided *p* < 0.05. The sample size is justified using the weighted kappa and degree of agreement.

## Results

**Figure 1:**
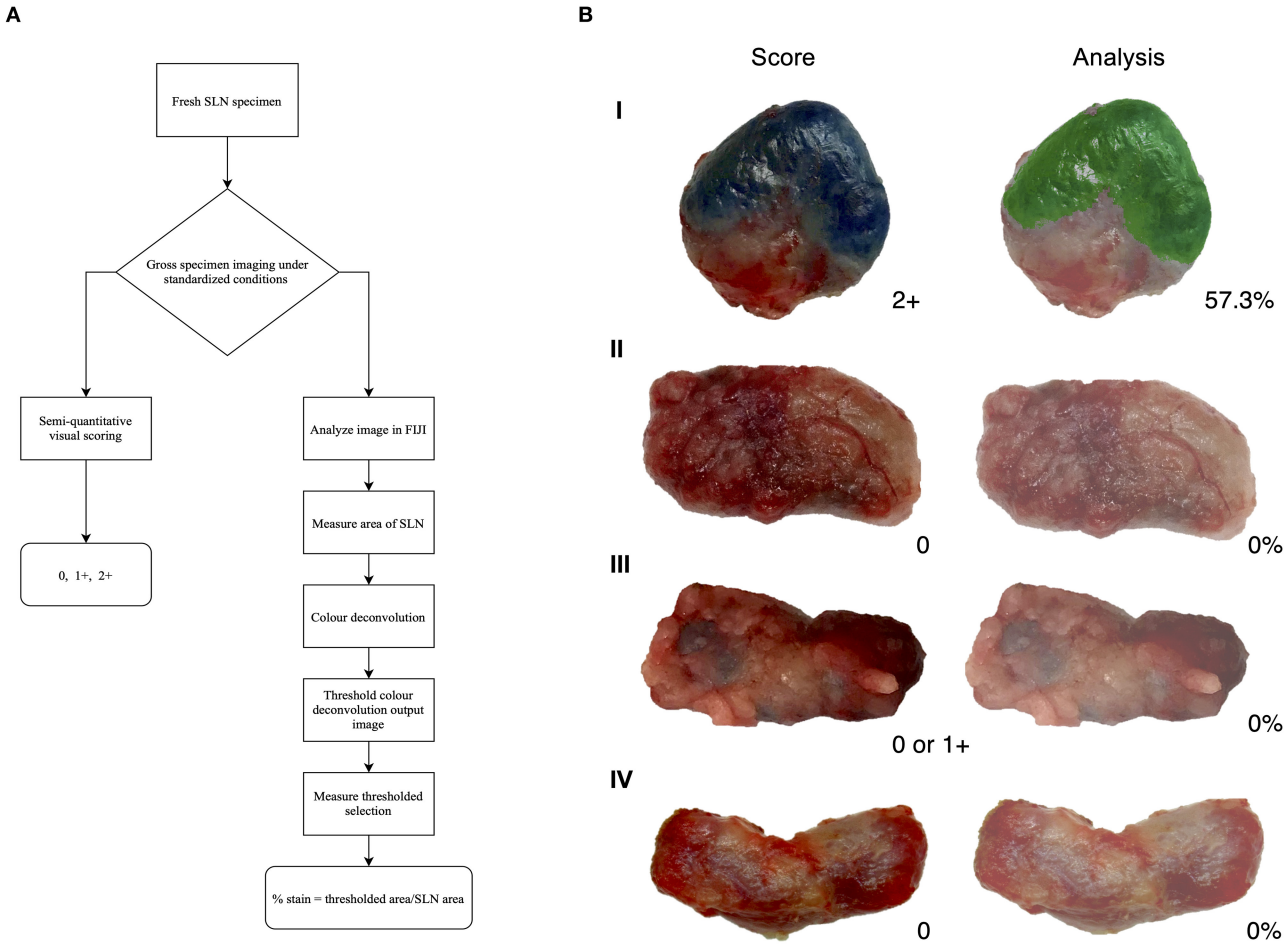
Visual assessment and image analysis outcomes. **(A)** Workflow of assessment for methylene blue stained lymph nodes. **(B)** Score and analysis of sentinel lymph nodes (SLN). Depicting a positive node (I), a negative node (II), an ambiguous node (III), and a negative control node (IV); all with agreement between score and analysis.

**Figure 2:**
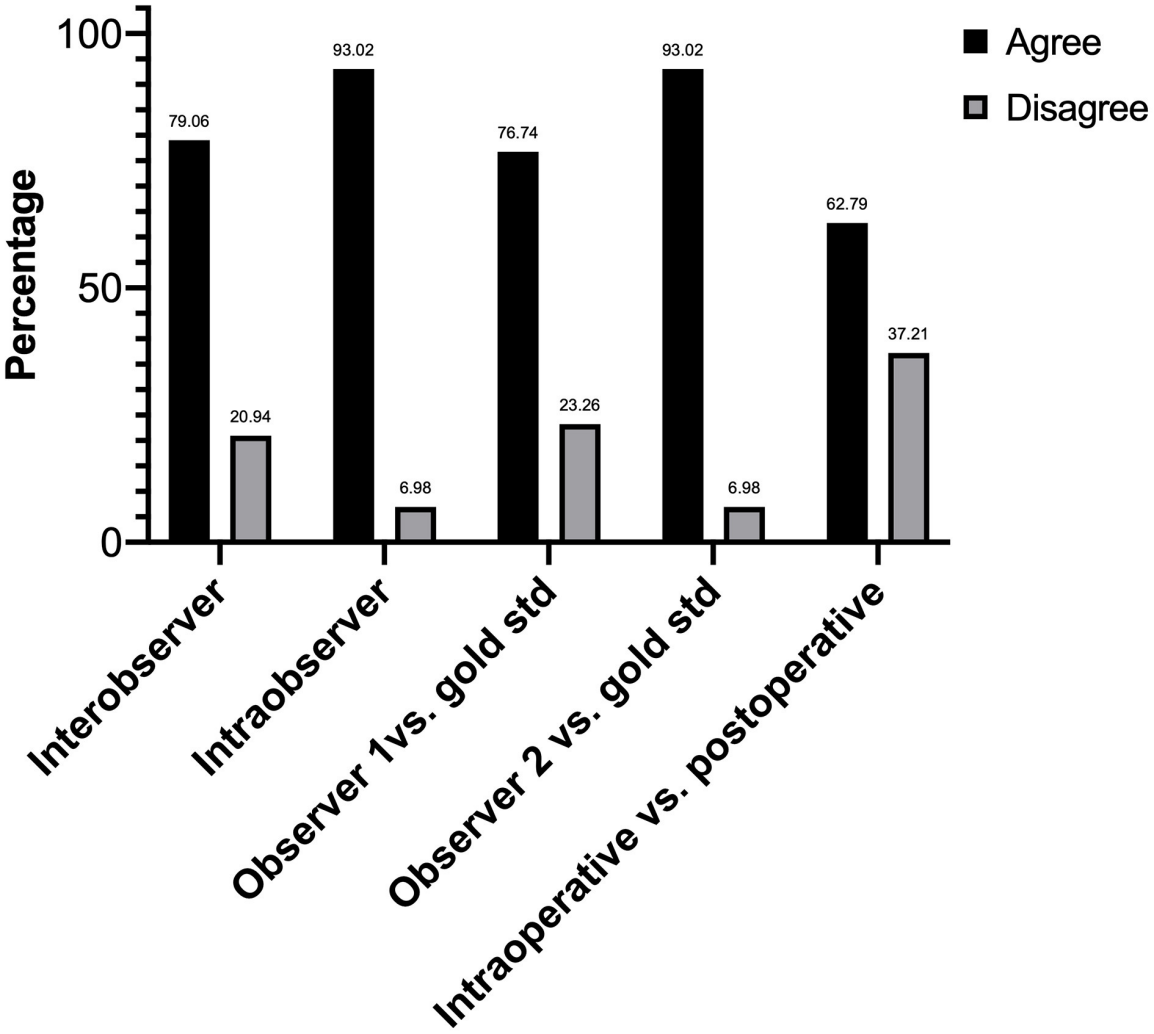
Histogram showing the frequencies of agreement and disagreement of scores in interobserver test, intraobserver test, comparing observers to the gold standard, and evaluating observer settings. All frequencies are statistically significant (*P* < 0.0001).

A total of 43 lymph nodes were collected from 25 clinical cases of patients undergoing lymph node extirpation with SLN mapping. Control lymph node images from three clinical cases were assessed during analysis and all depicted 0% of methylene blue staining (Supplementary Table 2). Lymph node images were sorted and analyzed as described in the methods (Figure 1A). A total of 33 images were included in the unstandardized group and 10 images in the standardized group. Distribution of visual assessment scores in different scoring settings (*in situ* intraoperative, postoperative *ex vivo*, and digital image) was evaluated (Table 1). Based on *ex vivo* visual assessment, 72% (31/43) of lymph nodes were scored as 1+ and 28% (12/43) categorized as 2+. Based on preliminary data, there is no significant difference in scoring based on standardized (κ = 0.8750, *p* = 0.0022) and unstandardized lymph node images due to excellent agreement (κ = 0.8097, *p* < 0.0001) between visual scores of images and scores based on image analysis (Supplementary Figure 1). Image analysis was successfully performed and documented in all lymph node images using ImageJ. There was significant concordance between the scoring system and image analysis (κ = 0.8581 [0.72–0.99], *P* < 0.0001; Table 2). Interobserver and intraobserver agreement of scoring was strong (κ = 0.7340 [0.58–0.89], *P* < 0.0001 and κ = 0.8983 [0.79–1], *P* < 0.0001, respectively). Observer 1 and observer 2 scoring compared to the gold standard displayed substantial agreement (κ = 0.6924, *P* < 0.0001 and κ = 0.8900, *P* < 0.0001, respectively). Evaluation of settings based on intraoperative and postoperative scoring displayed moderate agreement (κ = 0.5326 [0.39–68], *P* < 0.0001; Figure 2).

**Table 1:** Distribution of visual assessment scores observed intraoperatively, postoperatively, and with digital images.

**Score**	**Intraoperative**	**Postoperative**	**Digital image**
0	–^a^	28% (12/43)	30% (13/43)
1+	72% (31/43)	53% (23/43)	55% (24/43)
2+	28% (12/43)	19% (8/43)	14% (6/43)

a *Inclusion criteria of lymph nodes was based on blue staining assessed during intraoperative scoring, therefore scores of 0 were not included*.

**Table 2:** Frequency of agreement between visual scoring and analysis.

		**Quantitative image analysis**			**Total**
		**0**	**1+**	**2+**	
Semi-quantitative visual scoring system	0	10 (23.26)	2 (4.65%)	0 (0%)	12 (27.91%)
	1+	0 (0%)	23 (53.49%)	0 (0%)	23 (53.49%)
	2+	0 (0%)	2 (4.65%)	6 (13.95%)	8 (18.6%)
	Total	10 (23.26%)	27 (62.79%)	6 (13.95%)	43 (100.00%)
Percentage agreement	90.7%				
Weighted kappa	0.8581 [0.72–0.99]				

## Discussion

Sentinel lymph node mapping with vital dyes relies on accurate visual identification of lymph nodes thought to be “sentinel” based on tracer uptake and stains. Due to this subjective assessment, in human and veterinary medicine, the use of methylene blue alone is considered inferior in identification rates compared to radio-colloid imaging, fluorescence imaging or combined methods (18, 42). The poor identification rate results from the lack of sensitivity that the blue dye only technique presents (42). Therefore, it is imperative that accurate SLN mapping should not rely on blue dye alone and standardized evaluation methods for visualizing stain should be employed. Literature and research methodologies lack reporting and objective classification of lymph node staining obtained from SLN biopsies. Reports indicate identification rates (IR) as a point of success in the SLN mapping process (4), although appropriate criteria for successful identification of a SLN is often missing. Clinical trials involving SLN mapping outline inclusion criteria for SLNs reported based on nodes that are only stained blue (2, 7, 9, 18, 19, 43–49), blue and non-blue nodes with dye uptake in afferent lymphatic channels (12, 13, 15, 17, 50–55), or do not comment on IR inclusion criteria (32, 56–59). The inconsistent or lack of standardized, objective reporting across SLN mapping trials and cases that utilize methylene blue skews accuracy and reduces comparability of results between studies. Improving the reporting process of identified SLNs positive for methylene blue staining can influence the results of studies and enhance discernment of clinicians that assess these nodes.

In this report, we propose a simple, semi-quantitative visual scoring system to clinically assess methylene blue staining on lymph nodes extirpated during SLN mapping and validated the system using an image analysis process developed for quantification of stain. The assessment of staining is based on the amount of stain present on the surface of lymph node tissue. Our data shows a strong agreement between the scoring system and image analysis that was statistically significant. Immunohistochemistry (IHC) is the gold standard for visualization of antigens using antibodies and there are controlled, well-defined methods to quantify immunostaining patterns (60–62). Common histochemical stains also have validated quantification methods (39), however, this method is not developed to detect intraoperative methylene blue staining. Based on this, image analysis provides an objective, quick examination of tissue and can be used to improve histology process (63–67). The image analysis method demonstrated in this study was verified using images of negative control lymph nodes that did not pick up signals for stain and visibly true positive nodes that quantified all the methylene blue staining (Figure 1B). During image analysis, the color deconvolution (CD) method was employed due to the heterogeneity of lymph node tissue (29, 68). Color deconvolution allows the separation of RGB colors from images into stain channels made with specific vectors (36, 69) and this plugin is usually applied for the purpose of separating multiple histological stains in a tissue sample (70). The plugin produces a choice of vectors which are associated with specific dye mixtures. For the purpose of this study, the Giemsa vector was chosen. Giemsa is a dye that contains a mixture of methylene blue, eosin, and an optional third component, such as Azure B (70). Even though the Giemsa setting is for a combination of three stains, the methylene blue vector is verified and readily isolated (70). The output of the other channels will not show meaningful signals for stains, such as eosin. These stain channels are in grayscale and correspond to the intensity of a particular stain found in the image (69). This analysis plugin determines the density of stain in areas where multiples stains are co-localized (64). The parameters of 0–125 is a strict threshold based on controls to only detect dark pixels associated to methylene blue. Other studies, such as one by Onder et al. (36) have evaluated the robustness of the CD process and found that CD displayed significantly higher sensitivity in classification of stained samples without compromising specificity when compared to hue-saturation-intensity (HSI) separation method. Due to the superiority of CD in being able to detect dark areas that correspond to brown or blue which HSI could not differentiate, this plugin was employed in our image analysis. The three-grade scoring system developed in this study provides a simple semi-quantitative and accurate assessment of lymph nodes in a clinical setting and is consistent with existing scoring processes that are based on the overall stain intensity (i.e., percentage of cells stained) (63, 65, 71). The validation of our scoring system is based on IHC scoring methods that use image analysis to objectively validate the visual assessment (72–74).

Our scoring system has substantial interobserver agreement depicting there is low variance between scores given by observer 1 and observer 2. The low variability between two scorers illustrates that this visual assessment can be done by different observers and still yield the same score given to a sample. We hypothesize that the variability in scores between observers is seen when lymph node tissue coloration is ambiguous, if image quality is poor or due to differences in the learning curve (75, 76) to discern blue staining. The intraobserver agreement of scores in our study are near perfect and scores did not even vary when the blinded observer scored the randomized images in batches at a different time. Scores of different observers compared to the gold standard result in substantial agreement displaying that there is little variability between the score determined by individuals and that of the gold standard. Also, scoring between intraoperative and postoperative settings displayed moderate agreement. This depicts subpar congruency between each evaluation setting which may be attributed to the individual's learning curve toward ambiguous nodes or unstandardized settings, such as operating room low light, blood, or fat tissue impeding proper evaluation of blue staining. Further, the low agreement illustrates the need for *post-hoc* image analysis to verify ambiguous staining on nodes to prevent misidentification.

A strength of this study is the high degree of agreement that allows for the smaller sample size to be sufficient with high power. The image analysis program is practical and can easily be utilized by clinicians, researchers, and other hospital or lab staff since FIJI is an opensource, free software tailored for biosciences (35). The accessibility of the program can allow widespread use, allowing for limited variability in analysis programs between studies. Researchers may be less inclined to adopt this method of analysis if an expensive specialized software was required. Additionally, due to the use of a pre-existing, built-in plugin for deconvolution of stains and pigments in an image, the plugin contains verified vectors that correspond to specific stains, therefore validating the methylene blue stain detected in the images (35). Also, this visual assessment and image analysis program provides a short learning curve in appropriately scoring and analyzing the lymph nodes. Another strength this study poses is that the proposed method and workflow will allow for confidence and reproducibility in reporting, specifically when there are multiple investigators contributing to a study using methylene blue surgically. Whether, it be multiple researchers in the same facility or in different centers, using digital photos of lymph node specimens from all collected data pools we can more reliability report through *post-hoc* analysis whether the initial observer is correct in discerning a node as blue or not, as opposed to only relying on the observer's ability to make the assessment (31).

A limitation in this study can be the use of the visual scoring system in clinical practice. When encountering cases of lymph nodes with unstandardized settings, unusual lymph node morphology, ambiguous staining patterns, or differences in an individual's learning curve it could lead to discrepancies in visual scores between intraoperative and postoperative assessments. Cases such as these lower the agreement between intraoperative and postoperative visual scores. However, to overcome this challenge, *post-hoc* image analysis is an objective method to conclude on the staining status of the surgical specimen where analysis can adjust for unstandardized imaging conditions or detect faint “blue” signals. Another limitation of this study was the small sample size. In the concurrent study, patients were undergoing total lymph node basin extirpation to assess the accuracy of SLN mapping in that patient population. As a result, there are a large number of lymph node samples imaged that were negative for methylene blue stain (66.1%, 82/124) and not utilized for this study. Since the goal of this study was validation, we felt it was important to include only the lymph nodes identified as blue. Despite low numbers, the agreement coefficients were unaffected due to the analysis and scoring system having such high accuracy (Table 1) and as a result we were able to demonstrate excellent power in this study. Another potential limitation is the use of CD. Color devolution can have pitfalls in its ability to detect dark areas, where brown pigment can be falsely recognized as dark blue pixels (38, 69), usually when stains like diaminobenzidine (DAB) are used. This was seen with our original thresholds where SLNs scored as 0 are detected as a category of 1+ by image analysis since dark tissue is recognized as traces of blue. As a result, we were able to adjust our thresholds and consequently this was not a prominent issue due to the vector used and the strict thresholds. If the program does falsely recognize dark tissue as blue stain, the detection percentage is low and clinically negligible to the human eye where it ranges from 1 to 2% of stain detected. A final perceived limitation of the study may be the lack of automation to further the objectivity of the analysis process. An automated process for determining ROIs would be efficient and robust, however lymph nodes vary greatly in size and shape which make it difficult to tailor specific macros for ROI creation.

In conclusion, we developed a pragmatic visual and analytic assessment system to evaluate the degree of blue staining in extirpated lymph nodes when SLN mapping is performed using methylene blue dye. The scoring system and quantitative image analysis program have strong agreement which shows the validity of the visual assessment. This assessment workflow allows for standardized reporting of clinical research to improve comparability and consistency of results in SLN mapping of various cancers utilizing methylene blue. The validated visual scoring system provides an accessible and objective measure in a clinical setting when image analysis is not available. This visual scoring system can be utilized *ex vivo* in a patient-side manner, however, it is important to note that it is primarily intended for validation based on photographic imaging and analysis for standardizing reporting in research. It is not yet known if methylene blue staining patterns and uptake has significance for patterns of metastasis and outcomes, but a scoring and digital quantification system is required to investigate such research and is an objective of future directions.

## Data Availability Statement

The raw data supporting the conclusions of this article will be made available by the authors, without undue reservation.

## Ethics Statement

The animal study was reviewed and approved by Animal Care Committee, University of Guelph. Written informed consent was obtained from the owners for the participation of their animals in this study.

## Author Contributions

AR: conceptualization of ideas, conducting research, performing imaging, developed methodology, validating the experiment, conducted analysis, prepared the original manuscript draft, and completed further editing. KM and AS: critically reviewed and edited manuscript. JP: critically reviewed, edited manuscript, and provided oversight to the research activity. MO: conceptualization of research goals, performed surgeries and collection of lymph nodes, critically reviewed, edited manuscript, provided oversight to the research activity, and final approval of the version to be published. All authors contributed to the article and approved the submitted version.

## Conflict of Interest

The authors declare that the research was conducted in the absence of any commercial or financial relationships that could be construed as a potential conflict of interest.

## Publisher's Note

All claims expressed in this article are solely those of the authors and do not necessarily represent those of their affiliated organizations, or those of the publisher, the editors and the reviewers. Any product that may be evaluated in this article, or claim that may be made by its manufacturer, is not guaranteed or endorsed by the publisher.
