# Supplementary material for: Quantitative and Semi-quantitative Methods for Assessing the Degree of Methylene Blue Staining in Sentinel Lymph Nodes in Dogs
Source: Front Vet Sci. 2021 Oct 21;8:758295. doi: 10.3389/fvets.2021.758295 (PMC8567073; doi:10.3389/fvets.2021.758295)
Supplement: Supplementary file 1 [file Data_Sheet_1.pdf]

Supplementary Material

Table S1. Patient and tumor characteristics of cases for SLN mapping with methylene blue

Case	Breed	Sex	Tumor type	Tumor location	Lymphatic basin
634214	Golden Retriever	Fn	Mast cell tumor	Rostral maxilla	Submandibular, retropharyngeal
635020	Soft-coated Wheaten Terrier	Mn	Fibrosarcoma	Rostral maxilla	Submandibular, retropharyngeal
635960	Toy Poodle	Mn	Melanoma	Oral (gingiva)	Submandibular, retropharyngeal
636235	Mixed breed	Mn	Ameloblastoma	Oral (mandible)	Submandibular, retropharyngeal
637045	Mixed breed	Fn	Fibromatous epulis	Oral (mandible)	Submandibular, retropharyngeal
640555	American Bulldog	Fn	Carcinoma	Maxilla	Submandibular, retropharyngeal
641688	Soft-coated Wheaten Terrier	Fn	Fibrosarcoma	Oral (mandible)	Submandibular, retropharyngeal
644312	Labrador Retriever	Mn	Soft tissue sarcoma	Upper eyelid	Submandibular, retropharyngeal
644764	Mixed breed	Mn	Melanoma	Oral (upper lip)	Submandibular, retropharyngeal
645662	Labrador Retriever	Fn	Mast cell tumor	Perineum	Inguinal
645919	Golden Retriever	Mn	Mast cell tumor	Upper eyelid	Submandibular
646161	Cocker Spaniel	Fn	Carcinoma	Left ear	Prescapular, submandibular
647714	Golden Retriever	Fn	Carcinoma	Mammary gland	Inguinal, submandibular, retropharyngeal
650388	Labrador Retriever	Fn	Mast cell tumor	Right ear	Submandibular
650537	Boxer	M	Mast cell tumor	Scrotum	Inguinal, popliteal
651540	Giant Schnauzer	Fn	Melanoma	Right hindlimb 2nd digit	Inguinal, popliteal
652258	Mixed breed	F	Adenoma	Mammary gland	Inguinal
653456	Mixed breed	Mn	Carcinoma	Lung	Tracheobronchial
656637	Mixed breed	Mn	Fibrous hyperplasia	Maxilla	

654045	Mixed breed	Mn	Ameloblastoma	Oral	Submandibular, retropharyngeal
653832	Golden Retriever	Mn	Mast cell tumor	Left tricep	
652684	Standard Schnauzer	Mn	Melanoma	Nasal planum	Prescapular, axillary
651856	Miniature Schnauzer	Fn	Mast cell tumour	Left axilla	Prescapular, axillary
635056	Mixed breed	Mn	Mast cell tumour	Left axilla	Axillary
619299	Scottish Terrier	Mn	Sarcoma	Left forelimb	Prescapular
249770	Dandie Dinmont Terrier	Fn	Fibrosarcoma	Oral (cheek)	Submandibular, retropharyngeal
655515	Mixed breed	Fn	Renal cell carcinoma*	Left kidney	Retroperitoneal
655548	Mixed breed	Mn	Hematoma*	Spleen	Mesenteric, portal, splenic
657163	Chihuahua	F	Adenocarcinoma*	Thyroid gland	Submandibular, retropharyngeal

* These lymph nodes are methylene blue negative control cases.
Male (M); male neutered (Mn); female (F); female neutered (Fn)

Table S2. Image analysis of negative control lymph nodes

Lymph node[a]	Ex vivo score	Surface amount of MB stain[b]	Area of lymph node	Area of stain	Background
1	0	0	40875	0	0
2	0	0.807	143602	1159	0
3	0	0.0548	123984	68	0
4	0	0.108850942	14699	16	0
5	0	0.047326077	25356	12	0
6	0	0.003890142	25706	1	0
7	0	0.590364801	17955	106	0

[a] Specimens from 3 clinical cases
[b] Detection <1 is considered negligible and cause by image quality or light reflection.

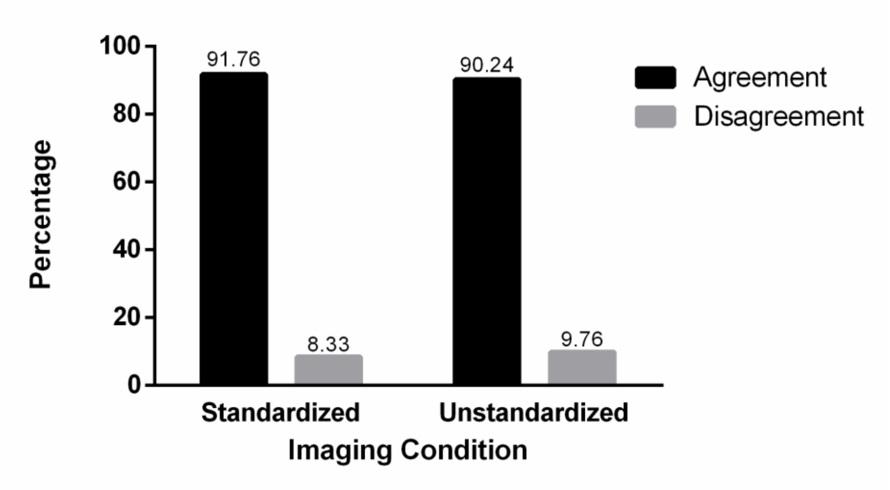

Figure S1. Percentage of agreement and disagreement of scores based on preliminary data. The scores from ex vivo visual assessment and scores based on image analysis of standardized images (N =12, $p = 0.0022$) and unstandardized images (N = 82, $p < 0.001$) were assessed. The number over the bar is the percentage of agreement or disagreement.
